# Examining Classic Bioimpedance Vector Patterns Between BMI Classifications Among Community-Dwelling Older Women

**DOI:** 10.3390/s25134181

**Published:** 2025-07-04

**Authors:** Kworweinski Lafontant, David H. Fukuda, Dea Chovatia, Cecil Latta, Chitra Banarjee, Jeffrey R. Stout, Rui Xie, Janet Lopez, Ladda Thiamwong

**Affiliations:** 1 Institute of Exercise Physiology and Rehabilitation Science, University of Central Florida, Orlando, FL 32816, USA; david.fukuda@ucf.edu (D.H.F.); jeffrey.stout@ucf.edu (J.R.S.); 2 College of Nursing, University of Central Florida, Orlando, FL 32826, USA; dea.chovatia@ucf.edu (D.C.); cecil.latta@ucf.edu (C.L.); chitra.banarjee@ucf.edu (C.B.); janet.lopez@ucf.edu (J.L.); ladda.thiamwong@ucf.edu (L.T.); 3 College of Medicine, University of Central Florida, Orlando, FL 32827, USA; 4 Disability Aging & Technology Cluster, University of Central Florida, Orlando, FL 32816, USA; 5 Department of Statistics and Data Science, University of Central Florida, Orlando, FL 32816, USA

**Keywords:** BIA, obesity screening, resistivity, impedance, bioelectrical

## Abstract

Body mass index (BMI) is not equipped to adequately detect obesity in individuals, leading to conditions such as normal-weight obesity, which disproportionately impact older women. Bioelectrical impedance vector analysis (BIVA) is a non-invasive and accessible method for assessing body composition and cellular health (e.g., resistance/height, reactance/height, phase angle), yet little is known about how BMI categories differ in cellular health. This cross-sectional study compared bioimpedance and adiposity across BMI classifications (normal weight, overweight, and obese) among 196 community-dwelling older women (age: 74.5 ± 7.0 years, BMI: 30.3 ± 6.3 kg/m^2^) using a one-way ANOVA and BIVA software. Individual and group bioimpedance were plotted within tolerance and 95% confidence ellipses. Body fat percentage (F = 70.6, *p* < 0.001, η_p_^2^ = 0.42) and resistance/height (F = 36.4, *p* < 0.001, η_p_^2^ = 0.27) differed between normal-weight, overweight, and obese groups. Reactance/height (F = 36.4, *p* < 0.001, η_p_^2^ = 0.27) and phase angle (F = 4.77, *p* = 0.01, η_p_^2^ = 0.05) only differed between normal-weight and obese groups. When plotted with 95% BIVA confidence ellipses, BMI categories occupied distinct positions from each other (T^2^ = 16.1 − 66.6, D = 0.68 − 1.48, *p* < 0.05). Within BIVA tolerance ellipses, overweight and obese categories fell in the “obese” quadrant, while the normal-weight category fell in the “athletic” quadrant. However, individual participants were predominantly scattered throughout the “cachectic,” “obese,” and “athletic” quadrants regardless of BMI. These findings suggest that BMI appears to be adequate for assessing population averages but not individual body composition. Future research should investigate the utility of bioelectrical resistance as a marker of obesity.

## 1. Introduction

The prevalence of obesity among older adults is a growing global epidemic [1]. Coupled with a natural decline in muscle mass with age, over 1-in-10 older adults globally live with sarcopenic obesity, a combination of sarcopenia and obesity characterized by an increased risk of disease and disability [2]. To combat these growing trends, clinicians first require an accurate assessment of obesity to better determine which older adults need medical and/or lifestyle interventions.

Body mass index (BMI) is the current standard for assessing obesity, with many key global health organizations using BMI as both the measure and definition of obesity [3]. Calculated as body weight in kilograms divided by height in meters squared, BMI is considered the superior anthropometric index for classifying individuals as underweight (<18.5 kg/m^2^), normal weight (18.5–24.9 kg/m^2^), overweight (25.0–29.9 kg/m^2^), and obese (≥30 kg/m^2^) [4]. However, when regressing body fat mass onto BMI, no more than half of the variance in body fat mass is accounted for by BMI [4]. Given that obesity is characterized by an excess of body fat mass, BMI may not be an adequate assessment of the body composition challenges faced by older adults [3,5].

Previous research on BMI has demonstrated poor diagnostic accuracy for identifying obesity among older adults when a traditional cut-off value of 30 kg/m^2^ is used [6]. This poor accuracy is more pronounced among older women, with only 19.6% being correctly classified compared with adiposity assessed by dual-energy X-ray absorptiometry (DEXA) [6]. These misclassifications may be due to variations in adiposity and muscularity that can exist among older women within a given BMI classification, particularly the normal-weight category [7,8]. There remains a need to identify clinically practical ways to objectively assess obesity in older women. This is an especially timely concern, given that a recent expert consensus statement on the definition and diagnostic criteria of clinical obesity called for the use of body composition assessments to confirm excess adiposity, limiting BMI to only a screening tool for suspected obesity and citing BMI’s high rate of misclassification [9]. The expert consensus statement also touched on the need for an accessible assessment, such as bioelectrical impedance analysis (BIA), to provide insight into the functional status and health of bodily tissues, which BMI fails to do [9].

**Figure 1:**
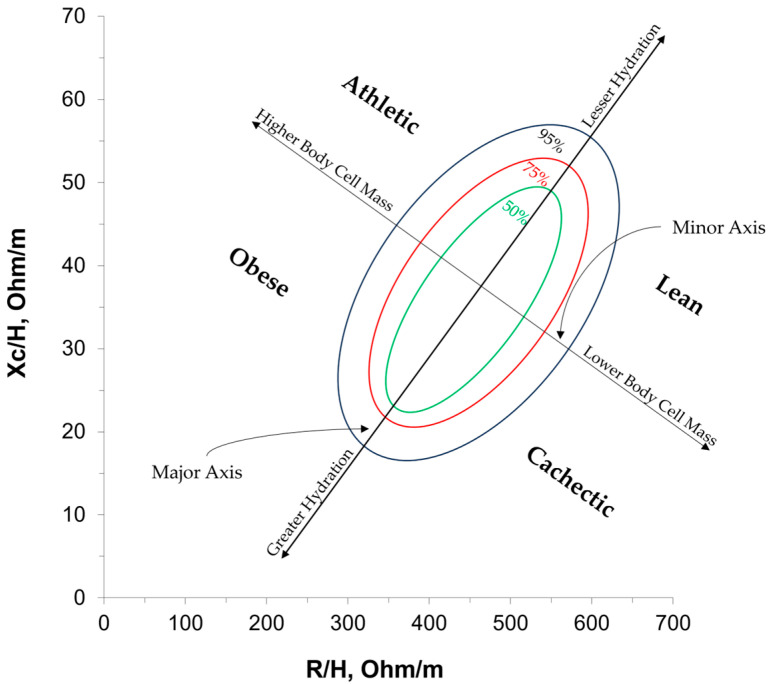
An annotated example of a bioelectrical impedance vector analysis (BIVA) graph with tolerance ellipses and qualitative quadrants. Individuals are plotted within this graph by their resistance (R) and reactance (Xc) at 50 kHz, and their position on the graph is compared with a similar reference population via tolerance ellipses (50%, 75%, or 90%) and quadrant labels, which have been established in previous research [10,19,20]. R/H, R/height; Xc/H, Xc/height.

BIA is a technique commonly used to estimate body composition via bioelectrical properties in the body, namely resistance (R), reactance (Xc), impedance (Z), and phase angle. R is the resistive property of cell membranes caused by total fluid volume and intra- and extracellular fluid distribution, observed as a decrease in voltage, and tends to have an inverse relationship with total fluid volume (i.e., total body water) [10,11,12]. Xc represents the inductive and capacitive properties of cell membranes and the surrounding tissue interfaces (i.e., dielectric properties) [10,11,12]. Z represents the overall opposition of a cell to an electrical current (i.e., the combination of R and Xc) [10,11,12]. Phase angle is the delay in an electrical current’s flow due to decreased cell membrane capacitance, accounting for R, Xc, and Z [13,14]. Phase angle is used as a measure of cellular integrity and a global indicator of cell membrane dysfunction [10,11,14,15,16]. Bioelectrical impedance vector analysis (BIVA) is a statistical extension of BIA and allows for the visualization of R, Xc, Z, and phase angle in a quantitative and qualitative manner [17], plotting individuals according to their R, Xc, Z vector length, and phase angle, while also classifying individuals into quadrants based on a demographically similar reference population [10,18,19,20], as shown in Figure 1. Previous research has typically labeled these quadrants as “athletic,” “lean,” “cachectic,” or “obese” to describe the characteristics of individuals in each quadrant compared with demographically similar peers [10,19,20]. BIVA can also be subdivided into either a classic or specific approach. The specific approach quantifies the volume of the body’s five conducting cylinders (i.e., limbs and trunk) through cross-sectional and circumferential measurements to standardize R and Xc. The classic approach standardizes R and Xc using the whole-body height (R/height and Xc/height), considering the entire body as a single cylinder. Despite their differences, both approaches have been shown to be sensitive to changes in nutritional and body compositional changes among older women [21,22]. Given the simplicity and time efficiency of forgoing cross-sectional and circumferential measurements, classic BIVA has the potential to grow in use as a quick assessment of obesity among older adults without the need for prediction equations.

Previous research has compared classic BIVA to BMI, demonstrating a positive association between BMI and phase angle up until 35–40 kg/m^2^ [23,24], as well as a negative association between BMI and vector length for individuals ranging from children to older adults [24]. Norman and colleagues compared BIVA variables between BMI classifications in adult men and women, reporting a significantly lower phase angle and higher R/height between the underweight and normal-weight categories [25]. However, this study was limited in generalizability as it focused on younger and older adults admitted into hospital care with benign gastrointestinal disease [25]. Other studies have used resistive and reactance indices (H^2^/R, H^2^/Xc, respectively) [26], compared specific BIVA between sarcopenic obese groups [27], and stratified BMI categories by age, race, and ethnicity [20], which all differ from the traditional classic BIVA approaches and limit the ability to compare findings.

BIVA may be able to improve clinical patient outcomes through the identification of obesity and other body composition challenges faced by older adults, such as sarcopenia, in line with the recent guidelines for defining and diagnosing clinical obesity [9]. Yet, it is currently unclear if BMI and BIVA classifications—both attempting to indicate potential health risks—differ among older women. Therefore, the purpose of this study was to examine potential differences in bioimpedance characteristics between BMI classifications among older women. Additionally, BIA estimations of BF% and skeletal muscle mass (SMM) were also compared between BMI categories as ancillary outcomes. We hypothesized that all bioimpedance characteristics would differ between BMI classifications.

## 2. Materials and Methods

### 2.1. Ethical Approval

This investigation was a preliminary analysis of an ongoing federally funded research study (NIMHD Grant R01MD018025), for which all methods have previously been reported [28]. All study procedures were approved by the University of Central Florida Institutional Review Board (STUDY00003206), pre-registered on ClinicalTrials.gov (NCT05778604), and conducted in accordance with the Declaration of Helsinki. All participants provided written informed consent prior to participation in this study.

### 2.2. Participants

We recruited 274 low-income community-dwelling older women from August 2022 to August 2024 for this cross-sectional investigation, comprising all female participants from the larger study. Participants were recruited through flier distribution, word-of-mouth, and community partners facilitating our introduction. Inclusion criteria were age > 60 years and low-income status based on the 2019 United States thresholds relative to family size [29], Individuals actively receiving treatment from a rehabilitation facility, as well as those with pacemakers, were excluded from this study. Additionally, participants with uncontrolled metabolic diseases/disorders were excluded from participation. After screening for inclusion criteria, 196 participants were included and analyzed in this study.

### 2.3. Bioelectrical Impedance Analysis (BIA)

BIA assessments were completed using an InBody s10 (InBody BWA, Audubon, PA, USA) phase-sensitive, direct segmental, multi-frequency device, which utilizes frequencies ranging from 1–1000 kHz. For whole-body Z, R, Xc, and phase angle, we established a standard error of measurement of 1.23 Ω, 1.23 Ω, 0.32Ω, and 0.03°, respectively, with our device using a subset of 15 similar older adults, measured twice back-to-back within the same session without repositioning. Additionally, the InBody s10 has demonstrated excellent test–retest reliability among older women in previous research [30]. The InBody s10 performed self-calibration upon start-up prior to testing for each participant.

**Figure 2:**
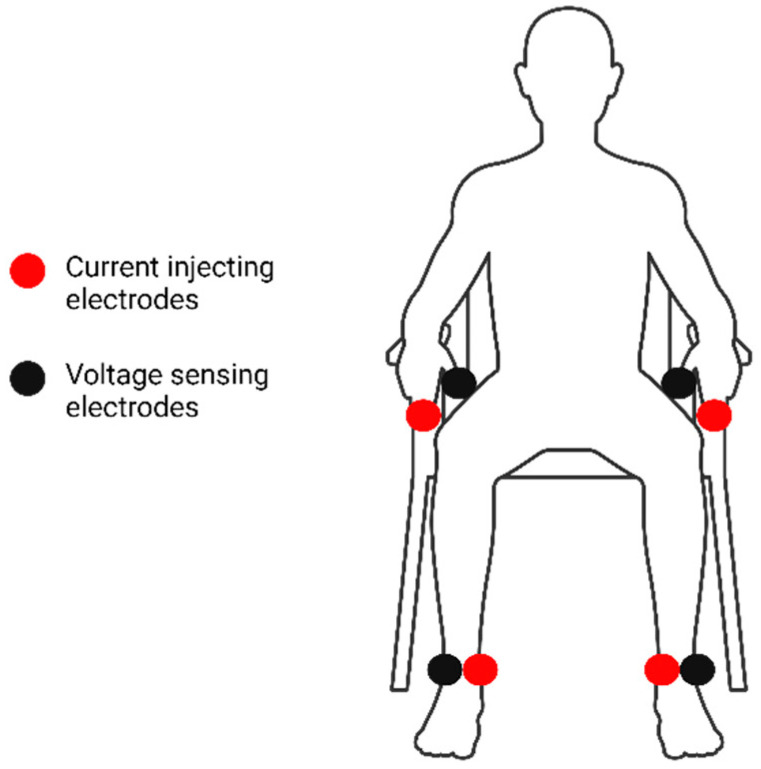
Locations of the InBody s10 current injecting and voltage sensing touch-type electrodes in the seated position [31]. Electrodes were placed on the left and right thumbs, middle fingers, and ankles, inferior to the malleoli.

Participants were instructed to arrive for a separate visit after initial screening and informed consent, having fasted for at least 3 h, abstained from caffeine and alcohol for at least 24 h, and avoided exercise for at least 6 h. Prior to testing, participants removed their shoes and metal jewelry. Height and weight were assessed using a digital physician scale and stadiometer (Health-O-Meter™, Model 402KL, McCook, IL, USA). Immediately after assessing height and weight, participants sat in a sturdy chair with armrests. The skin on their fingers and ankles was wiped using an InBody Tissue (InBody BWA, Audubon, PA, USA), and touch-type electrodes from the InBody s10 were placed on the right and left thumbs, middle fingers, and ankles (inferior to the medial and lateral malleoli), as shown in Figure 2. Participants remained seated and motionless for the 90 s duration of the BIA assessment. The seated position was used to increase accessibility among our older participants, as both prolonged standing and movement in and out of a supine position pose potential challenges for older adults. All body composition estimates (i.e., BF% and SMM) were derived directly from the InBody s10′s output and were ancillary outcome variables.

### 2.4. Statistical Analysis

This investigation was a preliminary analysis of baseline data from an ongoing study where the sample size justification was published previously [28]. All data were stored in a REDCap database managed by the University of Central Florida [32,33], and all statistical analyses were conducted using jamovi version 2.5.6 [34,35]. Normality of data was confirmed with Kolmogorov–Smirnov’s test. Levene’s test revealed unequal variances for Xc/height and SMM, so Welch’s one-way ANOVA with Games–Howell post-hoc comparisons was used for those variables. A one-way ANOVA with Tukey post-hoc comparisons was used for all other variables. Using BIVA software [36], individual bioimpedance characteristics were plotted with tolerance ellipses based on a reference sample of older women (N = 147, R/height = 399.0 ± 51.0 Ω/m, Xc/height = 32.0 ± 4.0 Ω/m, r = 0.41) assessed with a seca mBCA 515 (Seca GmbH & Co., KG, Hamburg, Germany) [37]. Mean bioimpedance characteristics were also plotted with 95% confidence ellipses for each BMI category, using Mahalanobis distance (D) to determine distances between each group mean and confidence ellipses and Hotelling’s T^2^ to determine Z vector length. Data were presented as mean ± standard deviation unless otherwise indicated. The threshold for statistical significance was set at *p* < 0.05. The sample size for this study resulted in an observed statistical power ranging from 0.58 (f = 0.18) to 1.0 (f = 1.22), calculated *a posteriori* using G*Power version 31 [38] with an alpha of 0.05.

## 3. Results

Table 1 provides characteristics of the included participants and results of the one-way ANOVA. None of the participants included were classified as underweight according to BMI (<18.5 kg/m^2^), so comparisons were only made between normal-weight, overweight, and obese categories. A one-way ANOVA revealed significant differences in age between groups (F = 4.03, *p* = 0.02, η_p_^2^ = 0.04; Table 1). To account for the potential moderating effects of age, we conducted an ANCOVA (Table 2).

Compared with the normal-weight group, overweight participants had significantly greater body mass (*p* < 0.001, d = −0.90) and BF% (*p* < 0.001, d = −0.92), but lower R/height (*p* < 0.001, d = 0.93). Compared with the normal-weight group, obese participants had significantly greater body mass (*p* < 0.001, d = −2.83), SMM (*p* < 0.001), BF% (*p* < 0.001, d = −2.08), and phase angle (*p* = 0.01, d = −0.53), yet lower R/height (*p* < 0.001, d = 1.54) and Xc/height (*p* = 0.002). Compared with obese participants, the overweight group had significantly greater body mass (*p* < 0.001, d = −1.92), SMM (*p* < 0.001), and BF% (*p* < 0.001, d = −1.16), yet lower age (*p* = 0.02, d = 0.47) and R/height (*p* = 0.001, d = 0.61). No other pairwise comparisons were statistically significant. Accounting for age via an ANCOVA did not alter Tukey or Games–Howell pairwise comparison outcomes.

**Figure 3:**
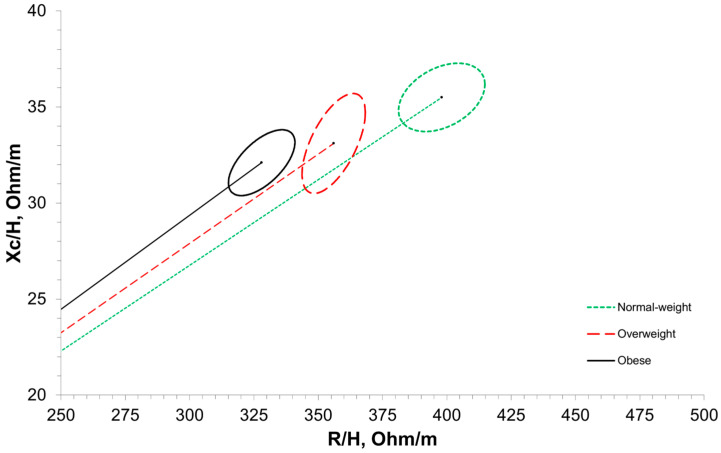
Bioelectrical impedance vector analysis (BIVA) with 95% confidence intervals around BMI classification averages. R/H, resistance/height; Xc/H, reactance/height. Normal weight = 18.5–24.9 kg/m^2^; overweight = 25.0–29.9 kg/m^2^; obese ≥ 30 kg/m^2^.

**Figure 4:**
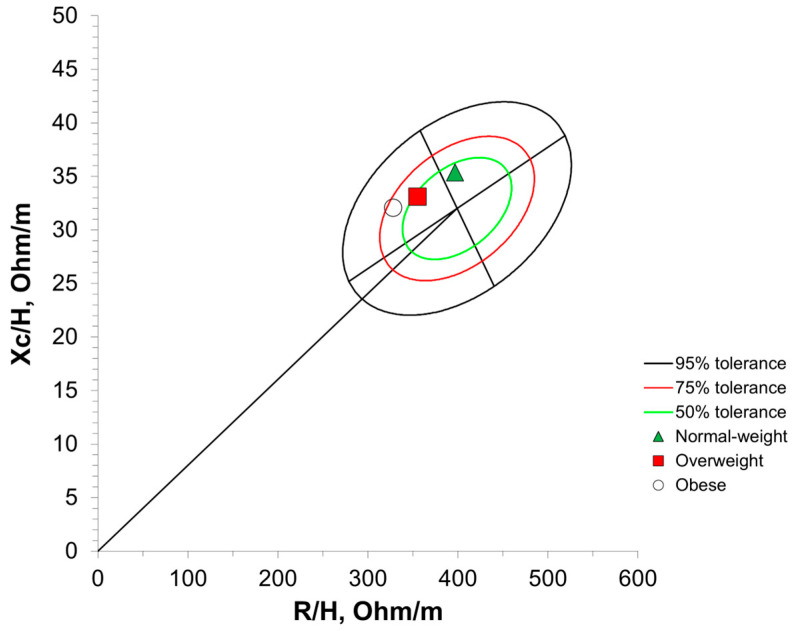
BMI categories distributed through a bioelectrical impedance vector analysis graph with tolerance ellipses. R/H, resistance/height; Xc/H, reactance/height. Normal weight = 18.5–24.9 kg/m^2^; overweight = 25.0–29.9 kg/m^2^; obese ≥ 30 kg/m^2^.

**Figure 5:**
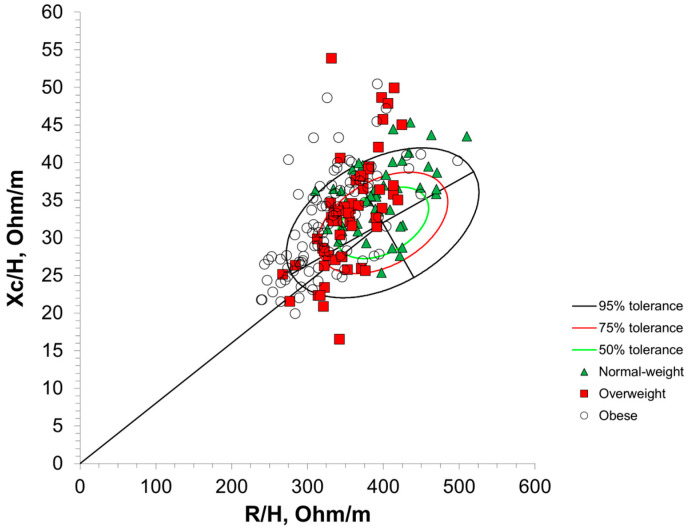
Individual participants categorized by BMI and distributed through a bioelectrical impedance vector analysis graph with tolerance ellipses. R/H, resistance/height; Xc/H, reactance/height. Normal weight = 18.5–24.9 kg/m^2^; overweight = 25.0–29.9 kg/m^2^; obese ≥ 30 kg/m^2^.

Figure 3 shows BIVA with 95% confidence intervals. The normal-weight group significantly differed in its vector length and confidence ellipse compared with the overweight group (T^2^ = 28.6, F = 14.2, D = 1.07, *p* < 0.001) as well as the obese group (T^2^ = 66.6, F = 33.1, D = 1.48, *p* < 0.001). The overweight group significantly differed in its vector length and confidence ellipse compared with the obese group (T^2^ = 16.1, F = 8.0, D = 0.68, *p* = 0.01). Figure 4 and Figure 5 present the distribution of the BMI groups and individual participants throughout the vector analysis graph with tolerance ellipses relative to a reference population [35].

## 4. Discussion

The purpose of this study was to compare bioimpedance characteristics and body composition between BMI classifications among older women. These results partially supported our hypothesis, with significant observed differences in BF%, R/height, and Z vector length (Figure 2) between normal-weight, overweight, and obese BMI classifications. However, both phase angle and Xc/height only differed between normal-weight and obese classifications.

Previous research by Norman et al. compared bioimpedance between BMI classifications among adults hospitalized with benign gastrointestinal disease, reporting no difference in phase angle between normal-weight, overweight, and obese individuals, despite significant Z vector migration between normal weight and obese as well as between overweight and obese [25]. Their results only partially align with the results of the present study, likely due to differences in the sampled populations. Phase angle has been well established as a global indicator of internal cellular health, sensitive to a host of cardiometabolic diseases and disorders such as cardiovascular disease and diabetes mellitus [13,40,41]. While obesity is considered a risk factor or comorbidity for several cardiometabolic conditions, BMI itself is not a direct measure of internal health and relies on inferences from anthropometry and body composition to gauge metabolic health [3]. This may explain the lack of difference in phase angle between normal-weight, underweight, and obese older adults observed by Norman et al., as BMI was not designed to classify individuals according to their internal health [4], and all participants in the study by Norman et al. homogenously had the same benign gastrointestinal disease [25]. This is additionally evidenced by the emergence of terms such as metabolically healthy obesity and normal-weight obesity, describing situations in which the BMI classification does not match the typical inference between relative weight and internal health [8].

The observed differences in R/height and Xc/height between BMI categories were consistent after controlling for age (Table 1 and Table 2). This indicates that differences in R/height and Xc/height between BMI categories are likely not age-related. That theory is supported by previous work in which Piccoli et al. compared BIVA confidence ellipses between BMI categories stratified by age, demonstrating a similar pattern of differences between BMI categories for all age groups [20]. While Norman et al. did observe significant differences in R/height between all BMI categories, they also observed significant differences in Xc/height between obese and overweight individuals [25]. The Xc of cell membranes is indicative of the capacity to store and release electrical currents, while R represents the resistive properties of cell membranes due to fluid shifts [10,12]. It is well understood that adipose tissue carries less water than SMM, and, given the conductive nature of water, this may explain the observed differences in R/height and lack of differences in Xc/height between BMI categories [42]. An increase in relative adiposity may decrease R, while an increase in relative muscularity may increase R [43,44]. Furthermore, the significant differences in R between BMI categories in the present study coincide with significant differences in BF%, supporting this theory.

**Figure 6:**
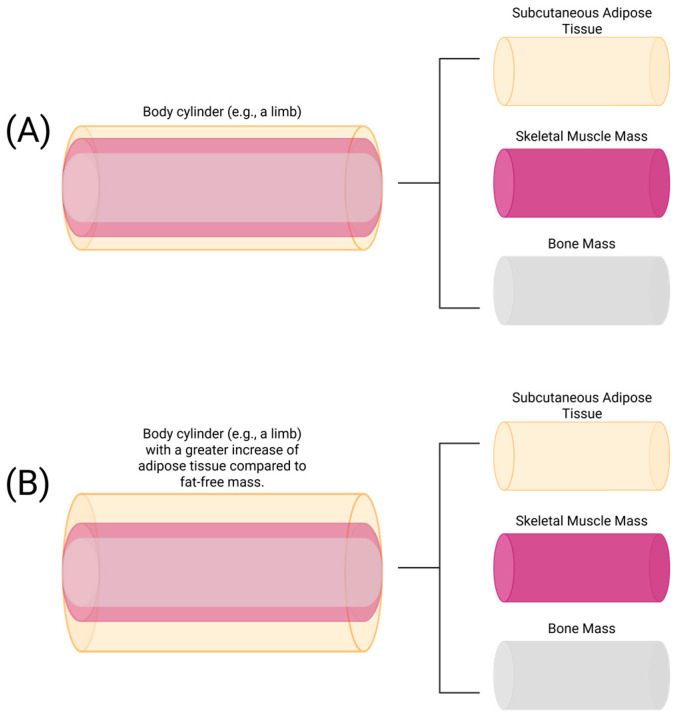
An illustration of the parallel tissue-resistor model [44], where the total resistance of a cylinder (e.g., a limb) equals the reciprocal sum of its sub-cylinders: subcutaneous adipose tissue, skeletal muscle mass, and bone. In (**B**), the size of the subcutaneous adipose tissue cylinder has increased while the size of the skeletal muscle mass and bone mass cylinders remained the same compared with (**A**), reducing total resistance of the cylinder, likely due to changes in fluid volume between the tissues. Created in BioRender. Lafontant, K. (2025). https://BioRender.com/f66v955, accessed on 3 July 2025.

Previous work by Baumgartner, Ross, and Heymsfield investigated the effect of obesity on bioelectric impedance within the parallel tissue-resistor model [44], first proposed by Rush, Abildskov, and McFee [43]. This model posits that the total R of a cylinder, such as a limb, subsumes the resistance of muscle, adipose tissue, and bone sub-cylinders, as seen in Figure 6 [44]. Based on this theoretical model, increasing levels of adiposity relative to muscle mass may reduce the ability for R to be used to accurately estimate fat-free mass [44]. For example, in previous research by Baumgartner, Ross, and Heymsfield, when the volume of adipose tissue was 1.6 times greater than the volume of skeletal muscle, a 6% overestimation in the volume of muscle mass was observed [44]. This demonstrates the sensitivity of R to increased fat mass relative to SMM (likely due to hydration differences between the tissue types) and may also help to explain the greater SMM observed in the obese participants compared with the normal-weight and overweight participants in the present study. If R is used to classify obesity, it may be able to circumvent issues with misclassifying athletes and those with excess SMM, which is a common issue with BMI [45]. Furthermore, normal-weight obesity, “skinny fat,” and sarcopenic obesity describe issues with BMI commonly faced by older women, where individuals classified as normal weight exhibit characteristics of metabolic syndrome due to a high amount of body fat relative to a low amount of muscle mass [8,46]. BIVA may be primed to detect normal-weight obesity and other BMI misclassifications due to muscle mass, given the effects of the adipose tissue-to-muscle mass volume ratio on R.

Within BIVA, classification into the obese category is largely dependent on a reduced R/height compared with the reference population, while the necessity for a reduced Xc to be classified as obese is largely dependent on the tilt of the major axis for the reference population [10,14]. Thereby, R/height may be an important indicator of obesity, although further research is needed. This is further corroborated by previous work from Brunani et al. and Norman et al., who both reported significantly reduced R/height in class III, II, and I obese individuals [25,47]. Through BIVA, the average position of each BMI classification was significantly different from other categories, despite some visual overlap in the 95% confidence ellipses of the obese and overweight classifications (Figure 3). However, the distribution of individual participants throughout the BIVA tolerance ellipses revealed variation, where individuals classified as obese, overweight, and normal weight via BMI were predominantly located in the athletic, obese, and cachectic quadrants (Figure 5). Only a few individual data points crossed into the lean BIVA quadrant, and they were normal-weight participants. When assessed by averages, BMI categories appear distinct and are in appropriate BIVA quadrants, with the normal-weight category residing in the athletic quadrant while the overweight and obese categories reside in the obese quadrant. Importantly, the quadrant labels may be misleading in this context, as this sample of older women may not be considered athletic by common standards; the R/height and Xc/height values should be the main points of interpretation for clinicians and researchers using BIVA rather than the quadrant labels. Nonetheless, these results are in line with the origins of BMI, as it was originally created to describe population averages and was not intended for individual assessments [4]. These results also further support the notion that BMI as a technique for the classification of individuals may not be the most accurate method available. Clinicians may be able to screen for obesity using BIVA while concurrently gaining information about cellular health via phase angle and hydration, thereby increasing the clinical practicality of this assessment.

While the present results show potential for R to detect relative increases in adiposity in a diverse sample of older women, there are limitations to be considered. The absence of an underweight group limits the ability to generalize the present results to individuals in that classification. Although the increase in adipose tissue volume relative to skeletal muscle volume has shown an ability to impact R [44], it is unclear if a decrease in adipose tissue volume, such as with underweight individuals, would have a similar effect. Future research should aim to target underweight individuals to expand upon the present results. We were unable to assess hydration status to ensure participants adhered to the pre-visit instructions prior to BIA assessments. However, previous research has indicated that violations of the pre-visit instructions relative to fasting and hydration may not lead to clinically significant changes in impedance assessments beyond the error of measurement [48]. Additionally, recommendations for the clinical application of BIA do not call for urine assessments prior to each BIA measure [49], as doing so would increase the cost (i.e., time and financial costs) and decrease the feasibility of BIA assessments in clinical practice. Our use of the seated position also aligned with feasibility in clinical practice, as some older adults may find movement in and out of the supine position or prolonged standing to be challenging. While comparisons of the present results to other research using supine or standing positions should be made cautiously [50,51], it is worth noting that there may not be a clinically relevant difference between postures; for example, Jensen et al. observed a 0.07° mean difference in phase angle between standing and seated postures among healthy adults, which was far less than the device precision of 0.5° reported in that study [50]. Therefore, the choice of posture in clinical practice should be one based on accessibility and preference.

The use of BIVA in clinical practice also necessitates the use of a reference population for the qualitative assessment of BIVA quadrants, despite the potential for inter-device and population sample differences [52]. The present study provides an older adult reference population for clinicians to use with the InBody s10 device, which may mitigate differences between BIA device models for clinicians who also use the InBody s10. Nonetheless, clinicians should be mindful of potential inter-device variations in bioimpedance measures and interpret our results with caution, as the observed results may have been different had a different BIA device been used [53]. With national epidemiological health surveys in countries such as the United States of America and Australia utilizing BIA [54,55], establishing bioimpedance cut-off values for obesity based on observed cardiometabolic comorbidities may reduce the need for reference population graphs, although more research is needed to test that theory.

## 5. Conclusions

While the ease of use and low financial barrier to BMI are undeniable, the quality of obesity screening and classification appears to be inadequate at the individual, but not group, level among older women. In contrast, BIA technology provides a nuanced assessment of body composition and appears to be sensitive to increases in adiposity relative to muscle mass through changes in R. Furthermore, BIA is becoming more accessible and affordable, with recent technological advances incorporating BIA into commercially available smartwatches and demonstrating validity in assessing body composition compared with lab-based criterion methods [56,57], although these smartwatches have yet to report R and Xc values directly. As the barriers to utilizing BIA continue to lower, more research is needed to determine if BIVA can be used in place of BMI to screen for obesity and provide clinicians with more information to inform their healthcare decisions than BMI can provide.

## Figures and Tables

**Table 1:** Participant characteristics (N = 196).

Variable	All (N = 196)	Normal Weight (n = 45)	Overweight (n = 56)	Obese (n = 95)
	Mean ± SD or n (%)	Mean ± SD or n (%)	Mean ± SD or n (%)	Mean ± SD or n (%)
Age (years)	74.5 ± 7.0	74.8 ± 7.3	76.4 ± 7.2 ^c^	73.1 ± 6.6 ^c^
BMI (kg/m^2^)	30.3 ± 6.3	23.0 ± 1.3	27.3 ± 1.5	35.5 ± 4.6
Body Mass (kg)	75.6 ± 17.7	57.2 ± 5.7 ^a,b^	67.4 ± 7.1 ^a,c^	89.1 ± 14.7 ^b,c^
BF%	40.2 ± 8.6	31.9 ± 7.5 ^a,b^	37.9 ± 5.8 ^a,c^	45.5 ± 6.4 ^b,c^
SMM (kg)	24.2 ± 5.0	21.2 ± 3.1 ^b^	22.6 ± 3.9 ^c^	26.5 ± 5.2 ^b,c^
Phase Angle (°)	5.4 ± 0.9	5.1 ± 0.7 ^b^	5.3 ± 1.0	5.6 ± 0.9 ^b^
R/height (Ω/m)	352 ± 52.9	398 ± 43.9 ^a,b^	356 ± 36.2 ^a,c^	328 ± 50.4 ^b,c^
Xc/height (Ω/m)	33.1 ± 6.7	35.5 ± 4.7 ^b^	33.1 ± 7.7	32.1 ± 6.7 ^b^
Race/ Ethnicity	AA: 77 (39.4%)	AA: 9 (20.0%)	AA: 14 (25.0%)	AA: 54 (56.8%)
	A: 13 (6.6%)	A: 5 (11.1%)	A: 7 (12.5%)	A: 1 (1.1%)
	H: 72 (36.7%)	H: 20 (44.4%)	H: 26 (46.4%)	H: 26 (27.3%)
	W: 31 (15.8%)	W: 9 (20.0%)	W: 9 (16.1%)	W: 13 (13.7%)
	O: 3 (1.5%)	O: 2 (4.5%)	O: 0 (0%)	O: 1 (1.1%)

BMI, Body Mass Index; SMM, skeletal muscle mass; BF%, body fat percentage; R, resistance; Xc, reactance; AA, African American; A, Asian; H, Hispanic; W, non-Hispanic White; O, other; SD, standard deviation; Statistical significance (*p* < 0.05) from the post-hoc pairwise comparisons is indicated as follows: normal weight = 18.5–24.9 kg/m^2^; overweight = 25.0–29.9 kg/m^2^; obese ≥ 30 kg/m^2^. ^a^ For normal weight compared with overweight. ^b^ For normal weight compared with obese. ^c^ For overweight compared with obese.

**Table 2:** One-way comparisons between normal weight, overweight, and obese categories (N = 196).

	Not Controlling for Age			Controlling for Age		
Variable	F	*p*-Value	η_p_^2^	F	*p*-Value	η_p_^2^
Body Mass	143.0	<0.001	0.60	139.1	<0.001	0.59
BF%	70.6	<0.001	0.42	69.6	<0.001	0.42
SMM	27.3	<0.001	0.22	24.2	<0.001	0.20
Phase Angle	4.77	0.01	0.05	3.34	0.04	0.03
R/height	36.4	<0.001	0.27	35.8	<0.001	0.27
Xc/height	4.07	0.02	0.04	7.56	<0.001	0.07

BF%, body fat percentage; SMM, skeletal muscle mass; R, resistance; Xc, reactance. Statistical significance (*p* < 0.05). Effect sizes are provided as partial eta squared (η_p_^2^), where 0.01, 0.06, and 0.14 represent small, medium, and large effect sizes, respectively [39].

## Data Availability

The data presented in this study are available upon request from the corresponding author, K.L. (ethical reasons).

## Abbreviations

The following abbreviations are used in this manuscript:

BMI	Body Mass Index
BIA	Bioelectrical Impedance Analysis
BIVA	Bioelectrical Impedance Vector Analysis
Z	Impedance
Xc	Reactance
R	Resistance
H	Height

## References

[1] Peralta M., Ramos M., Lipert A., Martins J., Marques A. (2018). Prevalence and trends of overweight and obesity in older adults from 10 European countries from 2005 to 2013. Scand. J. Public Health.

[2] Gao Q., Mei F., Shang Y., Hu K., Chen F., Zhao L., Ma B. (2021). Global prevalence of sarcopenic obesity in older adults: A systematic review and meta-analysis. Clin. Nutr..

[3] Fletcher I. (2014). Defining an epidemic: The body mass index in British and US obesity research 1960-2000. Sociol. Health Illn..

[4] Keys A., Fidanza F., Karvonen M.J., Kimura N., Taylor H.L. (1972). Indices of relative weight and obesity. J. Chronic Dis..

[5] Groothof D., Post A., Polinder-Bos H.A., Hazenberg B.P.C., Gans R.O.B., Bakker S.J.L. (2021). Muscle mass versus body mass index as predictor of adverse outcome. J. Cachexia Sarcopenia Muscle.

[6] Batsis J.A., Mackenzie T.A., Bartels S.J., Sahakyan K.R., Somers V.K., Lopez-Jimenez F. (2016). Diagnostic accuracy of body mass index to identify obesity in older adults: NHANES 1999–2004. Int. J. Obes..

[7] Shah N.R., Braverman E.R. (2012). Measuring adiposity in patients: The utility of body mass index (BMI), percent body fat, and leptin. PLoS ONE.

[8] Ormsbee M.J., Prado C.M., Ilich J.Z., Purcell S., Siervo M., Folsom A., Panton L. (2014). Osteosarcopenic obesity: The role of bone, muscle, and fat on health. J. Cachexia Sarcopenia Muscle.

[9] Rubino F., Cummings D.E., Eckel R.H., Cohen R.V., Wilding J.P., Brown W.A., Stanford F.C., Batterham R.L., Farooqi I.S., Farpour-Lambert N.J. (2025). Definition and diagnostic criteria of clinical obesity. Lancet Diabetes Endocrinol..

[10] Norman K., Stobaus N., Pirlich M., Bosy-Westphal A. (2012). Bioelectrical phase angle and impedance vector analysis—Clinical relevance and applicability of impedance parameters. Clin. Nutr..

[11] Ward L.C., Brantlov S. (2023). Bioimpedance basics and phase angle fundamentals. Rev. Endocr. Metab. Disord..

[12] Khalil S.F., Mohktar M.S., Ibrahim F. (2014). The theory and fundamentals of bioimpedance analysis in clinical status monitoring and diagnosis of diseases. Sensors.

[13] Lukaski H.C., Garcia-Almeida J.M. (2023). Phase angle in applications of bioimpedance in health and disease. Rev. Endocr. Metab. Disord..

[14] Baumgartner R.N., Chumlea W.C., Roche A.F. (1988). Bioelectric Impedance Phase-Angle and Body-Composition. Am. J. Clin. Nutr..

[15] Nescolarde L., Talluri A., Yanguas J., Lukaski H. (2023). Phase angle in localized bioimpedance measurements to assess and monitor muscle injury. Rev. Endocr. Metab. Disord..

[16] Lafontant K., Sterner D.A., Fukuda D.H., Stout J.R. (2024). A Non-Invasive Window into Cellular Health: Phase Angle and Impedance Ratio Explained. ACSMs Health Fit. J..

[17] Castizo-Olier J., Irurtia A., Jemni M., Carrasco-Marginet M., Fernandez-Garcia R., Rodriguez F.A. (2018). Bioelectrical impedance vector analysis (BIVA) in sport and exercise: Systematic review and future perspectives. PLoS ONE.

[18] Marini E., Buffa R., Saragat B., Coin A., Toffanello E.D., Berton L., Manzato E., Sergi G. (2012). The potential of classic and specific bioelectrical impedance vector analysis for the assessment of sarcopenia and sarcopenic obesity. Clin. Interv. Aging.

[19] Kyle U.G., Bosaeus I., De Lorenzo A.D., Deurenberg P., Elia M., Gomez J.M., Heitmann B.L., Kent-Smith L., Melchior J.C., Pirlich M. (2004). Bioelectrical impedance analysis—Part I: Review of principles and methods. Clin. Nutr..

[20] Piccoli A., Pillon L., Dumler F. (2002). Impedance vector distribution by sex, race, body mass index, and age in the United States: Standard reference intervals as bivariate Z scores. Nutrition.

[21] Fukuda D.H., Stout J.R., Moon J.R., Smith-Ryan A.E., Kendall K.L., Hoffman J.R. (2016). Effects of resistance training on classic and specific bioelectrical impedance vector analysis in elderly women. Exp. Gerontol..

[22] Rossini-Venturini A.C., Abdalla P.P., Fassini P.G., Dos Santos A.P., Tasinafo Junior M.F., Alves T.C., Gomide E.B.G., de Pontes T.L., Pfrimer K., Ferriolli E. (2022). Association between classic and specific bioimpedance vector analysis and sarcopenia in older adults: A cross-sectional study. BMC Sports Sci. Med. Rehabil..

[23] Di Vincenzo O., Marra M., Sacco A.M., Pasanisi F., Scalfi L. (2021). Bioelectrical impedance (BIA)—Derived phase angle in adults with obesity: A systematic review. Clin. Nutr..

[24] Buffa R., Mereu E., Comandini O., Ibanez M.E., Marini E. (2014). Bioelectrical impedance vector analysis (BIVA) for the assessment of two-compartment body composition. Eur. J. Clin. Nutr..

[25] Norman K., Smoliner C., Kilbert A., Valentini L., Lochs H., Pirlich M. (2008). Disease-related malnutrition but not underweight by BMI is reflected by disturbed electric tissue properties in the bioelectrical impedance vector analysis. Br. J. Nutr..

[26] Bosy-Westphal A., Danielzik S., Dorhofer R.P., Piccoli A., Muller M.J. (2005). Patterns of bioelectrical impedance vector distribution by body mass index and age: Implications for body-composition analysis. Am. J. Clin. Nutr..

[27] Marini E., Sulis S., Vorobel’Ová L., Stagi S. (2024). Specific bioelectrical vectors pattern in individuals with sarcopenic obesity. Clin. Nutr..

[28] Thiamwong L., Xie R., Park J.H., Lighthall N., Loerzel V., Stout J. (2023). Optimizing a Technology-Based Body and Mind Intervention to Prevent Falls and Reduce Health Disparities in Low-Income Populations: Protocol for a Clustered Randomized Controlled Trial. JMIR Res. Protoc..

[29] Poverty Thresholds. https://www.census.gov/data/tables/time-series/demo/income-poverty/historical-poverty-thresholds.html.

[30] Buckinx F., Reginster J.Y., Dardenne N., Croisiser J.L., Kaux J.F., Beaudart C., Slomian J., Bruyere O. (2015). Concordance between muscle mass assessed by bioelectrical impedance analysis and by dual energy X-ray absorptiometry: A cross-sectional study. BMC Musculoskel Dis..

[31] Lafontant K., Sterner D.A., Fukuda D.H., Stout J.R., Park J.-H., Thiamwong L. (2024). Comparing Device-Generated and Calculated Bioimpedance Variables in Community-Dwelling Older Adults. Sensors.

[32] Harris P.A., Taylor R., Minor B.L., Elliott V., Fernandez M., O’Neal L., McLeod L., Delacqua G., Delacqua F., Kirby J. (2019). The REDCap consortium: Building an international community of software platform partners. J. Biomed. Inform..

[33] Harris P.A., Taylor R., Thielke R., Payne J., Gonzalez N., Conde J.G. (2009). Research electronic data capture (REDCap)—A metadata-driven methodology and workflow process for providing translational research informatics support. J. Biomed. Inform..

[34] Love J., Droppmann D., Selker R., Gallucci M., Jentschke S., Balci S., Seol H., Agosti M. (2023). The Jamovi Project, Version 2.5.6. https://jamovi.org/.

[35] R: A Language and Environment for Statistical Computing. https://cran.r-project.org.

[36] Piccoli A., Pastori G. (2002). BIVA Software.

[37] Reljic D., Zarafat D., Jensen B., Herrmann H.J., Neurath M.F., Konturek P.C., Zopf Y. (2020). Phase Angle and Vector Analysis from Multifrequency Segmental Bioelectrical Impedance Analysis: New Reference Data for Older Adults. J. Physiol. Pharmacol..

[38] Faul F., Erdfelder E., Lang A.G., Buchner A. (2007). G*Power 3: A flexible statistical power analysis program for the social, behavioral, and biomedical sciences. Behav. Res. Methods.

[39] Cohen J. (2013). Statistical Power Analysis for the Behavioral Sciences.

[40] de Borba E.L., Ceolin J., Ziegelmann P.K., Bodanese L.C., Goncalves M.R., Canon-Montanez W., Mattiello R. (2022). Phase angle of bioimpedance at 50 kHz is associated with cardiovascular diseases: Systematic review and meta-analysis. Eur. J. Clin. Nutr..

[41] Jun M.H., Kim S., Ku B., Cho J., Kim K., Yoo H.R., Kim J.U. (2018). Glucose-independent segmental phase angles from multi-frequency bioimpedance analysis to discriminate diabetes mellitus. Sci. Rep..

[42] Thomas B.J., Ward L.C., Cornish B.H. (1998). Bioimpedance spectrometry in the determination of body water compartments: Accuracy and clinical significance. Appl. Radiat. Isot..

[43] Rush S., Abildskov J., McFee R. (1963). Resistivity of body tissues at low frequencies. Circ. Res..

[44] Baumgartner R.N., Ross R., Heymsfield S.B. (1998). Does adipose tissue influence bioelectric impedance in obese men and women?. J. Appl. Physiol..

[45] Grier T., Canham-Chervak M., Sharp M., Jones B.H. (2015). Does body mass index misclassify physically active young men. Prev. Med. Rep..

[46] Oliveros E., Somers V.K., Sochor O., Goel K., Lopez-Jimenez F. (2014). The concept of normal weight obesity. Prog. Cardiovasc. Dis..

[47] Brunani A., Perna S., Soranna D., Rondanelli M., Zambon A., Bertoli S., Vinci C., Capodaglio P., Lukaski H., Cancello R. (2021). Body composition assessment using bioelectrical impedance analysis (BIA) in a wide cohort of patients affected with mild to severe obesity. Clin. Nutr..

[48] Androutsos O., Gerasimidis K., Karanikolou A., Reilly J.J., Edwards C.A. (2015). Impact of eating and drinking on body composition measurements by bioelectrical impedance. J. Hum. Nutr. Diet..

[49] Kyle U.G., Bosaeus I., De Lorenzo A.D., Deurenberg P., Elia M., Manuel Gomez J., Lilienthal Heitmann B., Kent-Smith L., Melchior J.C., Pirlich M. (2004). Bioelectrical impedance analysis—Part II: Utilization in clinical practice. Clin. Nutr..

[50] Jensen B., Braun W., Both M., Gallagher D., Clark P., González D.L., Klückmann K., Bosy-Westphal A. (2020). Configuration of bioelectrical impedance measurements affects results for phase angle. Med. Eng. Phys..

[51] Roos A.N., Westendorp R.G., Frolich M., Meinders A.E. (1992). Tetrapolar body impedance is influenced by body posture and plasma sodium concentration. Eur. J. Clin. Nutr..

[52] Silva A.M., Matias C.N., Nunes C.L., Santos D.A., Marini E., Lukaski H.C., Sardinha L.B. (2019). Lack of agreement of in vivo raw bioimpedance measurements obtained from two single and multi-frequency bioelectrical impedance devices. Eur. J. Clin. Nutr..

[53] Bennett J.P., Cataldi D., Liu Y.E., Kelly N.N., Quon B.K., Gonzalez M.C., Heymsfield S.B., Shepherd J.A. (2024). Variations in bioelectrical impedance devices impact raw measures comparisons and subsequent prediction of body composition using recommended estimation equations. Clin. Nutr. ESPEN.

[54] Chumlea W.C., Guo S.S., Kuczmarski R.J., Flegal K.M., Johnson C.L., Heymsfield S.B., Lukaski H.C., Friedl K., Hubbard V.S. (2002). Body composition estimates from NHANES III bioelectrical impedance data. Int. J. Obes. Relat. Metab. Disord..

[55] Clifford S.A., Gillespie A.N., Olds T., Grobler A.C., Wake M. (2019). Body composition: Population epidemiology and concordance in Australian children aged 11–12 years and their parents. BMJ Open.

[56] Bennett J.P., Liu Y.E., Kelly N.N., Quon B.K., Wong M.C., McCarthy C., Heymsfield S.B., Shepherd J.A. (2022). Next-generation smart watches to estimate whole-body composition using bioimpedance analysis: Accuracy and precision in a diverse, multiethnic sample. Am. J. Clin. Nutr..

[57] Brandner C.F., Tinsley G.M., Graybeal A.J. (2023). Smartwatch-based bioimpedance analysis for body composition estimation: Precision and agreement with a 4-compartment model. Appl. Physiol. Nutr. Metab..

